# Vitamin D and ICU outcome in septic patients: a difficult connection?

**DOI:** 10.1186/cc13436

**Published:** 2014-03-17

**Authors:** FI Socci, A Cecchi, S Di Valvasone, M Ciapetti, L Perretta, ML Migliaccio, G Zagli, s Batacchi, G Cianchi, M Bonizzoli, A Terreni, A Peris

**Affiliations:** 1 AOU Careggi, Florence, Italy

## Introduction

Vitamin D, a secosteroid hormone, has roles in the optimal functioning of many organ systems and illnesses [1]. Recent reports show that most patients admitted to the ICU are vitamin D insufficient [2].

## Methods

In a 10-bed general ICU at the emergency department of a tertiary teaching hospital in Florence (Italy), 71 medical patients with severe sepsis or septic shock (51% men, 40% women) admitted to the ICU between January 2013 and September 2013 were studied. Vitamin D levels were measured by radioimmunoassay at admission, as well as demographic data (Table 1) and the Simplified Acute Physiology Score II (SAPS II). Exclusion criteria were: age <18 years, malnutrition state (body mass index (BMI) <18 kg/m^2^), pregnancy. Statistical analysis was carried out by linear regression and t test with SPSS 13. This study was approved by the Internal Review Board. Informed consent was obtained.

**Table 1:** Temperature measurement modalities used in EUROBACT intensive care units

	Age	SAPS	Length of stay ICU (days)	Length of stay in hospital (days)	Vitamin D (ng/ml)	BMI
**Mean**	62.59	52.48	10.14	24.90	11.35	26.38
**Median**	67.0C	54.00	6.00	20.00	9.60	24.83
**Mode**	77.00	40.00	3.00	8.00	8.00	24.69
**Standard deviatior**	17.85	18.35	10.17	20.70	7.16	8.33

## Results

Most patients showed vitamin D levels below 20 ng/ml, and we have not demonstrated a statistical significance correlation in the univariate regression between vitamin D level and both SAPS (*P *= 0.300) or length of stay in hospital (*P *= 0.154). Also our data do not demonstrate a statistical significance difference at t test between the value of vitamin D in the dead group and the alive group.

## Conclusion

Several groups have reported an inverse association between vitamin D levels in critically ill patients, severity of disease, outcome length of ICU stay and mortality [3,4]. In our experience we have not found an evident correlation between low vitamin levels and ICU outcomes. However, considering the prevalence of low vitamin D levels among the medical patients admitted to the ICU, further studies should be performed.
